# Functional products fortified with probiotic LAB isolated from Egyptian dairy sources showed hypolipidemic effects in Albino rats

**DOI:** 10.1371/journal.pone.0263241

**Published:** 2022-03-02

**Authors:** Amira M. G. Darwish, Marwa G. Allam, Enaam S. Shokery, Eman H. E. Ayad

**Affiliations:** 1 Arid Lands Cultivation Research Institute (ALCRI), City of Scientific Research and Technological Applications (SRTA-City), Alexandria, Egypt; 2 Food Science Dept., Faculty of Agriculture, Saba Basha, Alexandria University, Alexandria, Egypt; 3 Dairy Science and Technology Dept., Faculty of Agriculture, Alexandria University, Alexandria, Egypt; Government College University Faisalabad, PAKISTAN

## Abstract

Biological food industry has increased economic importance of new Lactic Acid Bacteria (LAB) with functional aspects such as health promoting and enhanced sensorial traits. The objectives of the present study were the isolation and genotypic characterization (16S rRNA) of new LAB isolates from Egyptian Laban Rayeb, Zabady and mothers’ breast milk as potential probiotics. Strains were *in vitro* and *in vivo* evaluated for their safety and probiotic health promoting traits in Albino rats then applied into two types of functional dairy products. Three strains *Streptococcus thermophilus* MH422542, *Enterococcus faecium* MH422543 and *Lactococcus lactis* subsp *lactis* MH422545, were selected from a pool of 82 strains. *St*. *thermophilus* showed proteolytic activity and production of Exopolysaccharides (EPS). When evaluated in animal models; the strains showed hypolipidemic effects especially *E*. *faecium* that reduced atherogenic indices up to 88%. The strains modulated the gut microbiota by reducing *Staphylococcus* sp. and coliforms with no adverse effects either on blood parameters, antioxidant enzymes, cancer markers or hepatocytes histological examination. Only *E*. *faecium* MH422543 showed an immune response by increased white blood cells (WBC) while lymph and platelets values were not affected. The probiotic strains approved their convenience as starter/ adjunct cultures and enhanced organoleptic properties of the fermented dairy products.

## Introduction

The development of probiotic food formulations is a key research area for the future functional food market. Economic forecasts expect the probiotic dietary supplements global market to increase from 3.3 up to 7 US$ billion between years 2015 and 2025 [1]. Different probiotics have shown to exert health-improving functional properties in most of probiotic species known as ‘‘core benefits” including regulation of intestinal transit, normalization of perturbed microbiota, competitive exclusion of pathogens, hypolipidemic, anti-atherogenic and antioxidant potentials [2, 3]. Other health benefits are probiotic strain specific e.g. immunological effects and production of bioactive compounds [4].

EPS-producing LAB has a greater ability to withstand technological stresses and survive the passage through the gastrointestinal tract (GIT) compared to the non-producing bacteria. *Streptococcus thermophilus* as a potential probiotic has been extensively used for the manufacture of several important fermented dairy foods, with number of functional activities such as production of extracellular polysaccharides, and demonstrated by various health effects, transient survival, and moderate adherence in the GIT and produce different metabolites with potentials beyond fermentation for lactic acid production [5]. On the other hand, when EPS are produced in situ, they impart highly desirable rheological changes such as; acting as natural biothickeners, giving the product a suitable consistency, improving viscosity, and reducing syneresis [6].

Proper *in vitro* studies such as acid and bile salt tolerance, should establish the stability and potential health benefits of probiotics prior to undertaking *in vivo* trials in order to ascertain that a given probiotic can achieve the desired purposes. As viability is a crucial key to achieve sufficient amounts until end of shelf life, probiotics need to be manufactured in such a way that they are robust and stable under the umbrella of good manufacturing practices and sanitary conditions to produce yoghurt of a wholesome quality [1, 7].

The objectives of the present study were the isolation, identification and characterization of new LAB strains isolated from Egyptian Laban Rayeb, Zabady and mothers’ breast milk, as potential probiotics. After genotypic identification, the isolates were evaluated *in vitro* and *in vivo* for their safety and probiotic health promoting. For guaranteeing maintenance of viability and acceptable organoleptic characteristics; selected probiotic strains were applied as starter or adjunct cultures in two types of functional fermented dairy products, in order to validate their convenience as probiotic starter cultures for functional fermented applications.

## Materials and methods

### Materials

Ten samples of Egyptian traditional Laban Rayeb and Zabady products were obtained from local markets in Alexandria Governorate, Egypt (31.205753 N 29.924526 E). Ten of healthy mothers’ milk samples were obtained from volunteers upon approval of the Institutional Committee of Faculty of Agriculture, Alexandria University, Egypt, who waived the need for their consents (31.23 N 29.95 E). Milk samples were collected between (1 October and 30 November, 2010). Growth media were M17 and MRS (Biolife, Italy), at 30, 37 and 42°C/ 24h. Pasteurized whole cow milk was obtained from the Dairy Pilot Plant, Faculty of Agriculture, Alexandria University, 3% fat and 8.5% Solids-Not-Fat (SNF). Reconstituted Skim Milk (RSM) "Davisco" (95% T.S., 1.2% fat and 32% protein) was obtained from Alexandria market. Yoghurt commercial starter culture Yo-Mix 495; *Streptococcus thermophilus* and *Lactobacillus delbrueckii* subsp. *bulgaricus* was obtained from Danisco Egypt.

### Isolation of lactic acid bacteria

For enrichment; 10 mL of each Rayeb and mothers’ milk samples were transferred into sterilized test tube, while one g of each Zabady sample was enriched in 10 mL sterilized reconstituted skim milk (RSM) (12.5% w/v) under aseptic conditions. The samples were incubated at 30°C, 37°C and 42°C for 24 h. Then, one mL of enriched milk was incubated in 10 mL appropriate broth media for 24 h before streaked on M17 agar (Biolife, Italy), incubated at 30°C and 42°C and MRS agar [8], incubated at 37°C and 42°C, for 24–48 h. Representative colonies were picked and incubated in appropriate broth media for 24h. After double checking and microscopic examination, the purified strains were stored in duplicates at (-20°C) in sterile reconstituted skimmed milk (RSM) (12.5% w/v) supplemented with 15% glycerol and registered in Faculty of Agriculture Saba Basha, Alexandria University Culture Collection (FABA).

### Phenotypic identification

Gram-positive and catalase negative purified cultures were stored at -20°C. Production of carbon dioxide, growth at 45°C, 10°C, in the presence of 6.5% salt, in pH 9.6 and in SF medium were tested. Biochemical characterization (carbohydrate fermentation), was performed as described by [9].

### Technological characterization

The acidification rate was measured by the change in pH during time in RSM at an appropriate temperature. The cultures were considered as fast, medium or slow-acidifying when a Δ pH of 0.4 units was achieved after 3, 3–5 and > 5 h, respectively as described by [10].

Individual strains were pre-grown in RSM containing 0.1% yeast extract for 6 h, 1% of each culture was added to 100 mL skimmed Ultra-high temperature (UHT) milk, sensory evaluation was carried out after incubation at an optimum temperature. The intensity of flavor was scored on a scale from (1–4) 1, slightly; 2, moderate; 3, strong; 4, very strong and the overall grade of 10 according to [11].

For autolytic activity assessment, the isolates were pre-grown in optimum broth media and temperature for 24 h, and then re-inoculated at 30°C, 37°C and 42°C to follow the growth at 0, and after 3, 6, 24 and 48 h by measuring optical density at 650 nm (OD_650_). The autolytic activity was determined as the percentage decrease in the absorbance at 650 nm at different time intervals according to [12].

For proteolytic activity examination; the overnight pre-grown cultures were streaked on prepared plates of sterile RSM and plate count agar medium (PCA) (Biolife, Italy) with a ratio of 3:7, examined for any halo of proteolysis around according to [13]. Antibacterial activity was determined using agar well-diffusion assay against Enteropathogenic *E*. *coli* obtained from the culture collection of NIZO (Food Research, Ede, The Netherlands) as described by [14].

For Exopolysaccharides (EPS) production investigation, the isolates were activated in M17 broth for 24 h, streaked on M17 agar medium then incubated at 37°C for 24 h. At the end of incubation, screening test was carried out for mucoidy of colonies determined by visual appearance, and rope length was determined by touching them with a sterile inoculation loop (The inoculated loop method) [15, 16] strains were considered positively slime producer if the length of slime was above 1.5 mm.

### *In-Vitro* assessment of potential probiotic LAB strains

Acid tolerance was tested on pH (2.0, 3.0 and 4.0 ±0.1) adjusted with HCl while bile salt resistance was tested using MRS broth containing 0.2, 0.3 and 0.4% (w/v) bile salts (Biolife, Italy); measuring optical density at 650 nm (O.D_650_) using a spectrophotometer (Spectronic 20D+, U.S.A.) as described by [17]. Cultures growth development in each broth was compared with MRS broth (pH 6.6) control.

Based on previous results three strains were selected; RM732, ZP7411 and HT741 for genotypic characterization and further analyses.

### Genotypic characterization of selected LAB isolates

Bacterial isolates were grown, harvested, DNA was collected then the DNA pellets were dissolved in 50 μl nuclease free water and were kept in -20°C according to [18]. The full length of the 16S rRNA gene was amplified by polymerase chain reaction (PCR) [19] using the universal primers 27 F (5-AGAGTTTGATCCTGGCTCAG-3) and 1492 R (5-GGTTACCTTGTTACGACTT-3) were performed as described by [20]. The PCR products were checked on 1% agarose as described by [21], purified prior to sequencing by using PCR Clean Up Promega Kit. The DNA sequences of PCR products were determined by using an AB 373 DNA sequence (Applied Biosystem, Mubarak City for Scientific Research). Basic local alignment search tool (BLAST) algorithm was used to search for homologous sequences in GenBank through the World Wide Web of GenBank; National Center for Biotechnology Information (NCBI) **http://www.ncbi.nlm.nih.gov**. Software Bioedit was used to align the query with other sequences in the Genbank then phylogenetic trees of the bacterial isolates were drawn. The partial sequences of 16S rRNA were deposited to the GenBank database under the accession numbers MH422542 (*Sreptococcus thermophilus* RM732), MH422543 (*Enterococcus faecium* ZP7411) and MH422545 *Lactococcus lactis* subsp *lactis* HT741).

### *In vivo* assessment of potential probiotic LAB strains

Twenty male albino rats (70±10 g) were bred and maintained in a colony at Physiology Department, Faculty of Medicine, Alexandria University, Egypt after the approval of Alexandria University Ethical Committee (AlEXU-IACUC), a member of International Council for Laboratory Animal Science (ICLAS) (Permission number: AU08200415366). All experiments were performed in accordance with Alexandria University Ethical Committee guidelines and regulations. Animals were housed in stainless steel wire-mesh cages in a room maintained at 22±1°C. Rats were acclimatized fed laboratory chow (with chemical composition as follows; fat 2.8%, protein 18.5%, fiber 11.2%) and water ad libitum for 1 week to stabilize their metabolic condition. After adaptation week, the rats were randomly divided into four groups of five animals each, Group (G1); the control group fed the commercial chow and drank pasteurized milk (3% fat, 8.5% SNF). Groups (G2), (G3) and (G4); were fed the commercial chow and drank cultured milk prepared with initial inoculation level 10^8^ CFU mL^-1^ of selected single strains; *St*. *thermophilus* MH422542, *E*. *faecium* MH422543 or *Lactococcus lactis* subsp *lactis* MH422545 respectively, for five weeks.

At the end of experiment (6 weeks), final weights were recorded, and rats were sacrificed after overnight fasting under isoflurane 5% anesthesia according to [22]. Blood samples were collected in EDTA tubes for blood analyses and plasma, sodium heparin tubes for plasma analyses. Immediately after necropsy, the rats’ organs; liver, kidneys, brain and spleen were dissected out carefully and weighed. The rats’ livers then fixed in 10% formol saline at 4°C for 48 h, embedded in paraffin blocks, sectioned and stained for histological light microscopic evaluation according to [23]. Hematology parameters were determined using (Cell-Dyn® 6000 Hematology analyzer), lipid profile and Carcinoembryonic Antigen (CEA) were determined with (Hitachi 7600 Biochemistry Autoanalyzer) and antioxidative enzymes (SOD and TBARS) were determined using Enzyme-linked immunosorbent assay (ELISA) (Sigma-Aldrich, Merck KGaA) [24]. Atherogenic indices formula were calculatedas follows; Friedwald formula, LDL /HDL [25] and another index which calculated as LDL /Total Ch for confirming results [26].

The microbiological analyses of probiotic cultures in large, small intestines and fecal population were held; for counting the lactobacilli group; the MRS agar pH 5 (Biolife, Italy), was used for 48 h at 37°C. For counting the cocci LAB the M17 agar (Biolife, Italy), was used for 48 h at 37°C. *Staphylococcus* spp. was enumerated on Staph 110 media (Biolife, Italy), for 48 h at 37°C. Coliforms were counted on Violet Red Bile agar (Biolife, Italy), at 37°C for 20 h according to [26].

### Application

#### Cultured milk beverages

Pasteurized milk (11.5% TS) was wormed to 42°C and cultured with 1–2% of individual isolated potential probiotic LAB strains; *St*. *thermophilus* MH422542, *E*. *faecium* MH422543 or *Lactococcus lactis* subsp *lactis* MH422545 (CM1, CM2 and CM3 respectively) with inoculation level of 10^8^ CFU mL^-1^ (based on preliminary experiment), The mix was distributed in bottles, incubated at 42°C/ 3 h, then cooled and stored at 4°C.

#### Probiotic yoghurt products

Cow milk was standardized using skimmed milk powder (SMP) to SNF (13%). The milk was homogenized and heat-treated at 85°C/ 15 min. Milk was cooled to 42±1°C, divided into four equal portions and inoculated with individual isolated probiotic strains; *St*. *thermophilus* MH422542, *E*. *faecium* MH422543 or *Lactococcus lactis* subsp *lactis* MH422545 for (Y1, Y2 and Y3 respectively) an hour before inoculation with (0.03 g/kg) of commercial yoghurt starter Yo-Mix 495, while control was inoculated directly with Yo-Mix 495. The inoculated milk was then poured into 100 mL plastic cups and incubated at 42±1°C until set coagulation at pH ~4.6 (About 5 h.), then cooled and stored at 4°C [27].

#### Microbiological and chemical analyses

Counting and detecting viability of LAB probiotic strains was monitored on MRS agar (Biolife, Italy), and potato dextrose agar (Biolife, Italy) for counting yeast and molds, as recommended by [28].

Titratable acidity was expressed according to [29], pH was determined, acetaldehyde content as mg/ 100 g was determined using the basic Fuchsine reagent according to [30].

#### Sensory evaluation

The sensory evaluation of cultured milk beverages and probiotic yoghurt fresh samples were assessed by 20 consumer-oriented panellists (8 men and 12 women, 27 to 51 years) at Food Science Department, Faculty of Agriculture, Alexandria University, Egypt according to [31–33]. The criteria for selection depended on their experience and background related to fermented dairy products. The representative samples which had not damaged or changed during cold storage at (4˚ C), were allowed to rest at room temperature (25˚ C) 10 min before evaluation. After panellists individual orientation, they were allowed to evaluate the samples independently using a 10-point Hedonic scale [34].This scale consisted of the test parameters of taste, odor, texture, appearance and overall acceptability, accompanied by a scale of ten categories as: 1 = dislike extremely; 2 = dislike much; 3 = dislike moderately; 4 = dislike slightly, 5 = neither dislike nor like, 6 = like slightly; 7 = like moderately; 8, 9 = like much; 10 = like extremely. Panellists were instructed to rinse their mouth with water between samples to eliminate any residual effects.

### Statistical analysis

The data were presented as mean values ± standard deviation. Statistical analysis was performed using one-way analysis of variance (ANOVA) followed by Duncan’s test. The differences were considered significant at (*p* < 0.05) using IBM SPSS Statistics 23 software program for statistical analyses (IBM Corp (2015) IBM SPSS Statistics for Windows, Version 23.0. IBM Corp, Armonk, NY).

## Results and discussion

### Isolation, identification and characterization of LAB isolates

After isolates were phenotypically identified (morphologically and biochemically), seven Rayeb, 16 Zabady and 59 mothers’ milk isolates were selected for next stage of examinations.

The flavor formation of wild isolated LAB strain in milk culture is the main pillar of qualifying these strains for industrial use [35]. *St*. *thermophilus* MH422542 showed remarkable ability of producing fruity Rayeb-like flavor, *E*. *faecium* MH422543 was described as fruity citrus while *Lactococcus lactis* subsp *lactis* MH422545 showed Ester Karish-like flavor.

Table 1, illustrates technological properties of the three selected isolated strains. All strains showed slow acidification ability. In spite of that the slow rate of acid development may promote undesirable side effects which can adversely affect the quality of yoghurt [27], but on the other hand, slow acidifying strains was reported to be one of the effective approaches of inhibiting yoghurt post-acidification during cold storage [36]. Additionally, *In situ* produced EPS from LAB can enhance acid gel properties of fermented dairy products via both ropy and cell-bound EPS [6].

**Table 1:** Technological properties of selected isolated strains.

Strain	identification	Acidification	EPS	%Autolytic	Proteolysis
		Activity		Activity	(+; -)
MH422542	*St*. *thermophilus*	Slow	+	5.17±1.30^a^	+
MH422543	*E*. *faecium*	Slow	-	4.08±0.93^b^	-
MH422545	*L*. *lactis* subsp *lactis*	Slow	-	2.09±0.84^c^	-

Data represented are means ±SD.

^a,b,c,…^Means values in the same column marked with unlike letters are significantly different (*p*<0.05).

MH422542, *Streptococcus thermophilus* RM732; MH422543, *Enterococcus faecium* ZP7411; MH422545, *Lactococcus lactis* subsp *lactis* HT741.

The obtained results emphases the EPS producing strain, Rayeb strain MH422542 (*St*. *thermophilus*), which nominated its selection for further applications. EPS producing ability by *St*. *thermophilus* was previously reported [37–39]. All strains showed weak autolytic activity, while *St*.*thermophilus* MH422542 strain showed proteolytic activity. Proteolytic activity of *St*.*thermophilus* was previously indicated [5]. Results negates presence of antimicrobial compounds in neutralized cell-free supernatants used against *E*. *coli*, but generally the preservative role of these strains cannot be denied through producing sufficient amounts of organic acids shown in their acid producing ability [40].

### *In vitro* assessment of potential probiotic LAB

**Fig 1:**
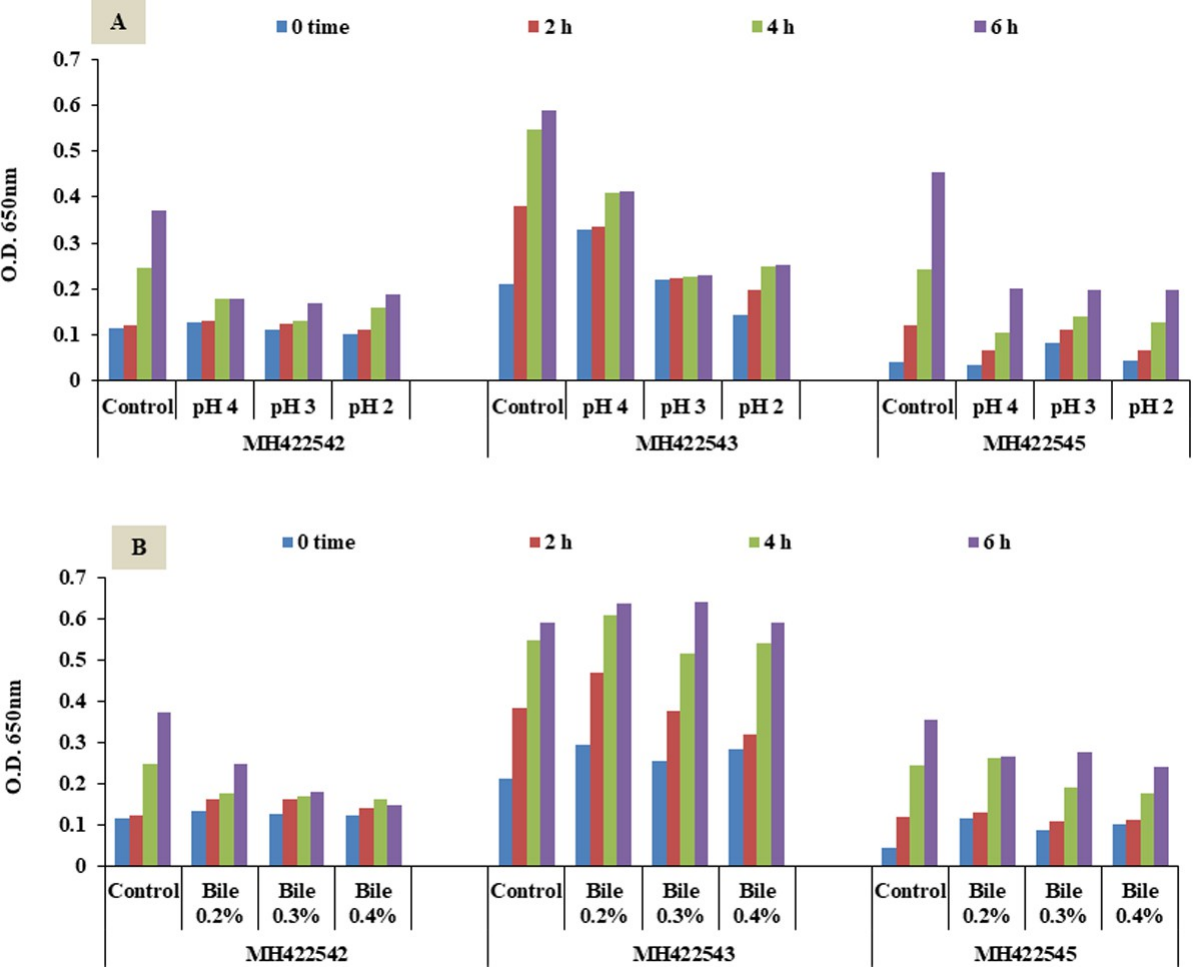
*In vitro* assessment of strains’ acid and bile resistance. A, Acid tolerance; B, Bile resistance.

Acid and bile resistance *in vitro* assessment of strains; *St*. *thermophilus* MH422542, *E*. *faecium* MH422543 and *Lactococcus lactis* subsp *lactis* MH422545 (Fig 1), revealed their acid and bile salt tolerance which allowed their selection for further analyses. Probiotics should withstand the adverse conditions encountered in the upper (GIT), these attributes are strain dependent and should be taken into account to serve the selection of more stable probiotic strains for human use [41].

### Molecular identification (16S rRNA)

**Fig 2:**
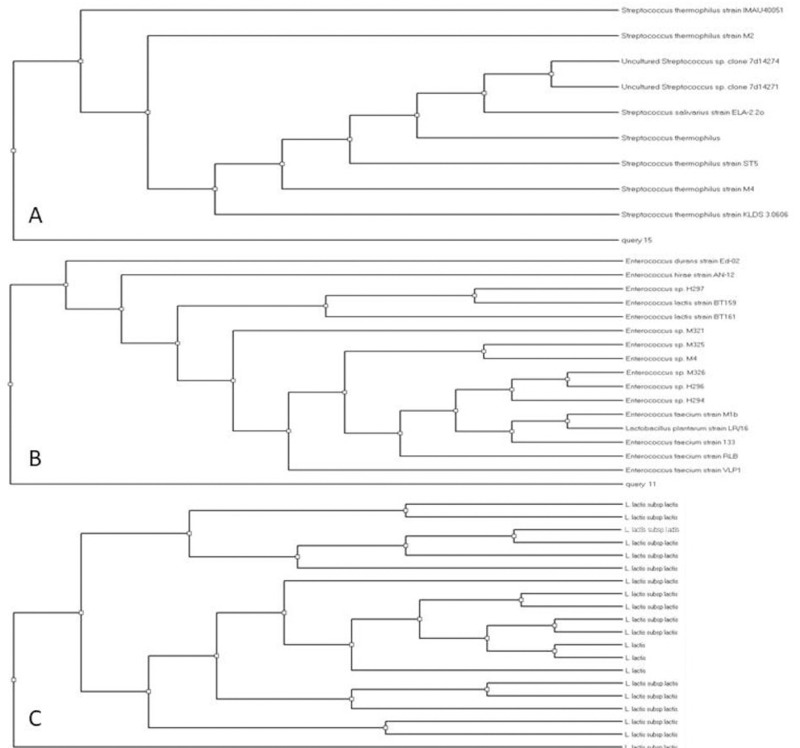
Phylogeny tree of partial sequences of the three selected isolates. A, MH422542 (*Streptococcus thermophilus*); B, MH422543 (*Enterococcus faecium*); C, MH422545 (*Lactococcus lactis* subsp *lactis*).

Depending on the technological characterization and probiotic potentials results; 16S rRNA sequencing approach was employed to identify the three selected probiotic LAB strains; RM732, ZP6411 and HT741. Amplified 16S rRNA genes were partially sequenced using DNA sequencer. The results of BLAST (basic local alignment search tool) analysis was used to draw Phylogeny trees (Fig 2A–2C). Multiple alignments were carried out to confirm the results of Blast analysis, then the partial sequences data of the three probiotic strains were deposited to the GenBank Data Library under the accession numbers; MH422542 (*St*. *thermophilus* RM732), MH422543 (*Enterococcus faecium* ZP7411) and MH422545 (*Lactococcus lactis* subsp *lactis* HT741).

### *In vivo* assessment of potential probiotic LAB strains

**Fig 3:**
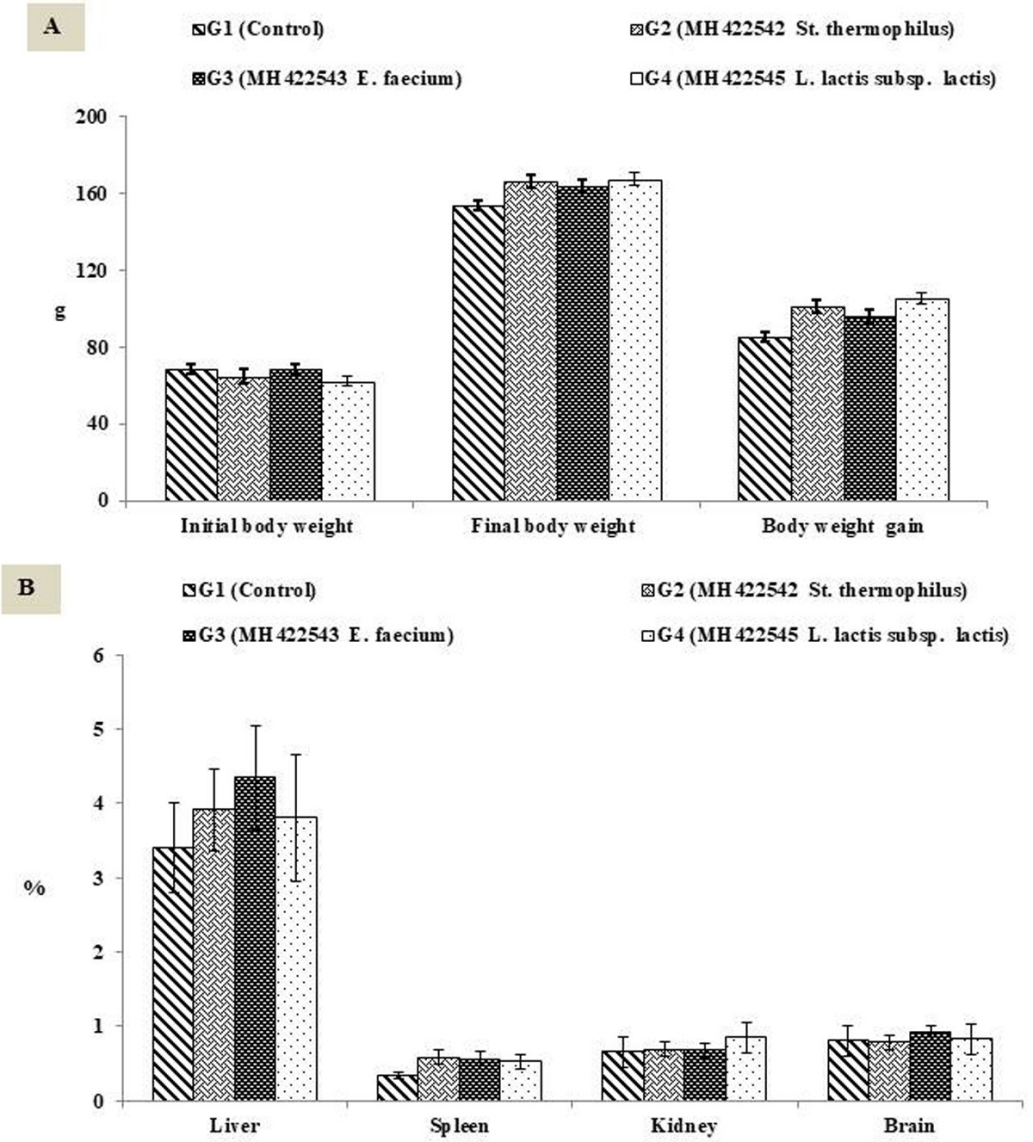
Body weight gain and relative organs’ weights of treated Albino rats. A, Body weight gain; B, relative organs’ weights (expressed as a percentage of body weight). Data represented are means of (n = 5) rats ±SD.

Fig 3A and 3B exhibits the body weight gain and relative organs’ weights of treated animals. All animals remained healthy for the duration of the study. Feeding rats with cultured milk increased body weight at the end of the experiment but did not affect the relative organs’ weights (g organ per 100 g body weight), these results agreed Zommara [26].

**Fig 4:**
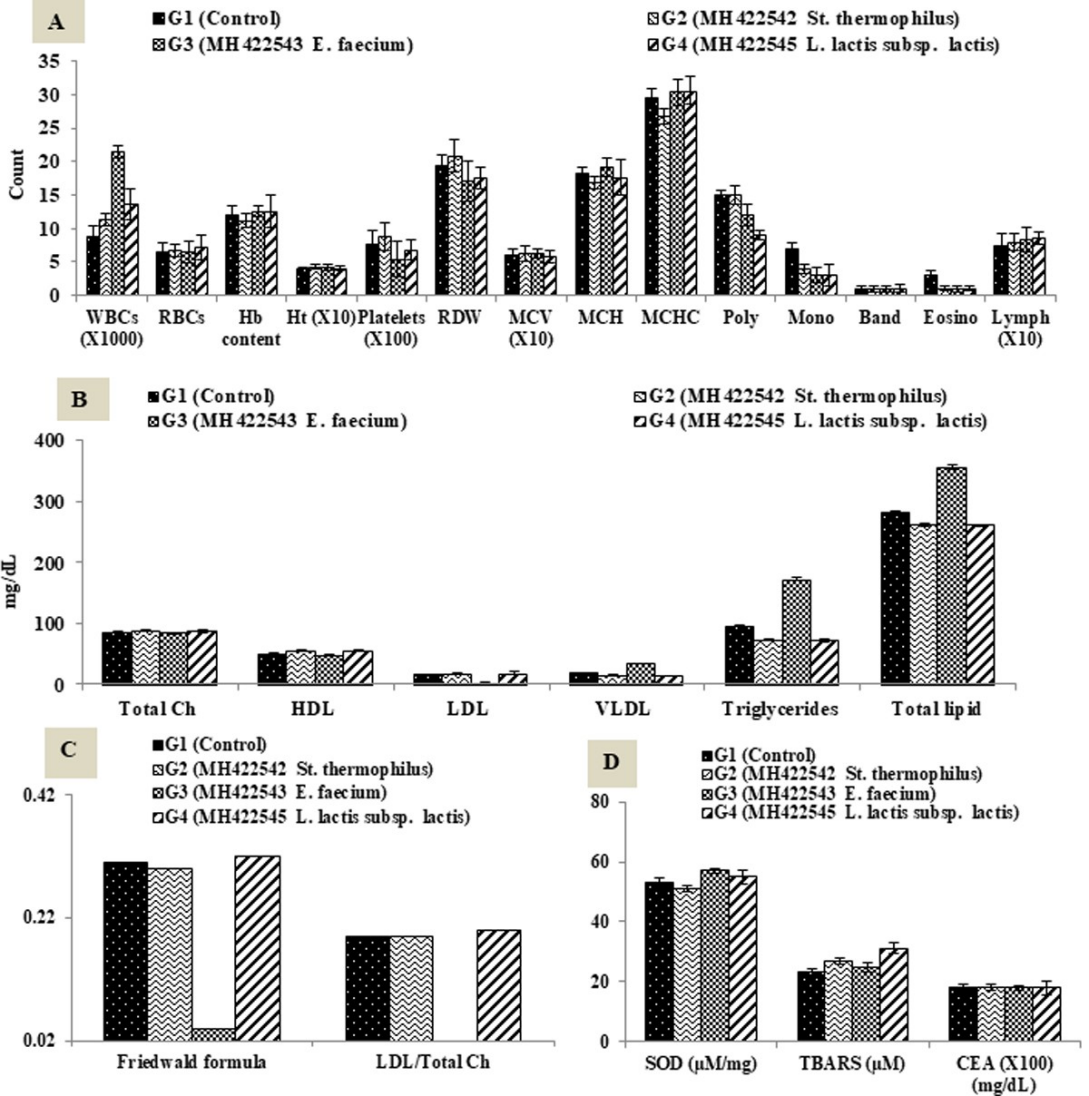
Effect of ingested probiotics on blood biochemical parameters. A, Complete blood counts CBC; B, Plasma lipid profile; C, Atherogenic indices; D, Antioxidant enzymes and Carcinoembryonic antigen (CEA). X1000, X100 and X10 indicates that actual values have been multiplied X times. Data represented are means of (n = 5) rats ±SD. Ch, Cholesterol; HDL, High Density Lipoprotein; LDL, Low Density Lipoprotein; VLDL, Very Low Density Lipoprotein; TG, Triglycerides; SOD, Superoxide Dismutase; TBARS, Thiobarbituric acid reactant substances; CEA, Carcinoembryonic antigen.

Hematology parameters results illustrated in Fig 4A–4D. Fig 4A showed an increase of WBCs count accompanied with increase lymphocytes in group G3 which fed *E*. *faecium* MH422543, that could indicate an immune response as long as lymph and platelets values were not affected, as previously reported [42, 43].

Fig 4B of plasma lipid profile showed that the ingested strains showed a hypolipidemic effect in different ways; although *E*. *faecium* strain showed higher concentrations in triglycerides (TG) and total lipids; but its hypolipidemic effect was expressed by remarkable suppression in LDL concentrations and reduction in atherogenic indices up to 88%. Atherogenic indices (Fig 4C) have more than one formula which are more sensitive than measurement of total cholesterol (TCh) as predictor (or for follow-up) of coronary heart disease and cardiovascular risk [25, 44]. On the other hand, the dietary groups fed *St*. *thermophilus* and *Lactococcus lactis* subsp *lactis*; G2 and G4 respectively tended to have hypolipidemic effect lowering TG and total lipids concentrations. Hypolipidemic effects of lactic cultures have been reported earlier by several workers [42, 45, 46].

Superoxide dismutase (SOD) is a major antioxidant enzyme and one of the first line defence antioxidants; that dismutates superoxide to hydrogen peroxide increasing antioxidative status [47, 48]. Lipid peroxidation of unsaturated fatty acids is a frequently used indicator of increased oxidative stress and subsequent oxidative damage [49], which is very commonly detected by the measurement of thiobarbituric acid reactant substances (TBARs) as an end-product.

Results illustrated in Fig 4D showed the no effect of ingested probiotics on oxidative stress of treated rats represented in SOD or TBARs. Probiotics administration was reported to ameliorate oxidative stress status [50, 51].

The carcinoembryonic antigen (CEA) test is the most frequently used test in human serum to monitor the amount of this protein that may appear in the blood in certain kinds of cancers [52]. Results revealed that CEA was less than 0.2 (mg/dL) in all groups including control group, which indicates the safety of the tested probiotic strains.

**Fig 5:**
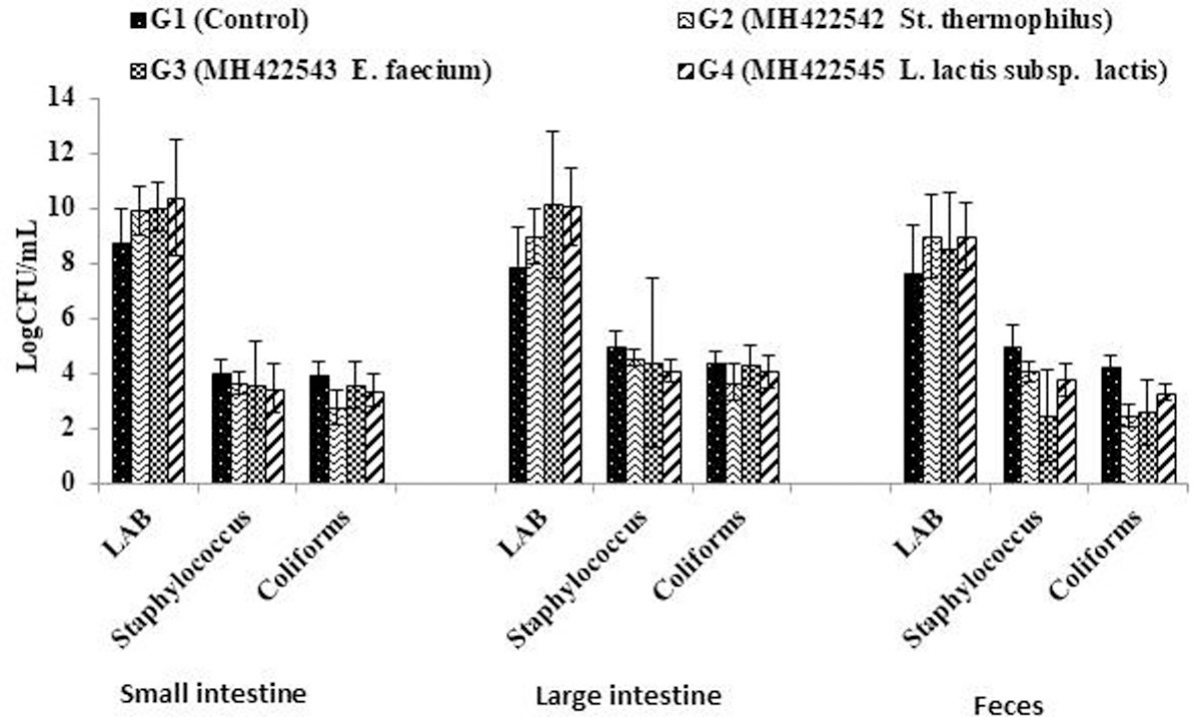
Influence of probiotic cultures on intestinal microbiota and fecal population. Data represented are means of (n = 5) rats ±SD.

The impact of probiotic cultures on; small and large intestines as well as their recovery from feces for the three strains, in addition to their inhibition role on naturally inhabitant pathogens such as *staphylococcus* spp. and coliforms is exhibited in Fig 5. The results showed that the small and large intestinal microbiota in treated rats groups exhibited increase in LAB counts comparing to control as well as in feces. All rats groups received cultured milks exhibited reduction in *Staphylococcus* sp. and coliforms counts compared to control. Modulation of the gut microbiota through the inhibition role of LAB probiotics against *Staphylococcus* sp. and coliforms may be relayed to metabolic compounds produced by LAB, which have antimicrobial effects [45, 53, 54].

**Fig 6:**
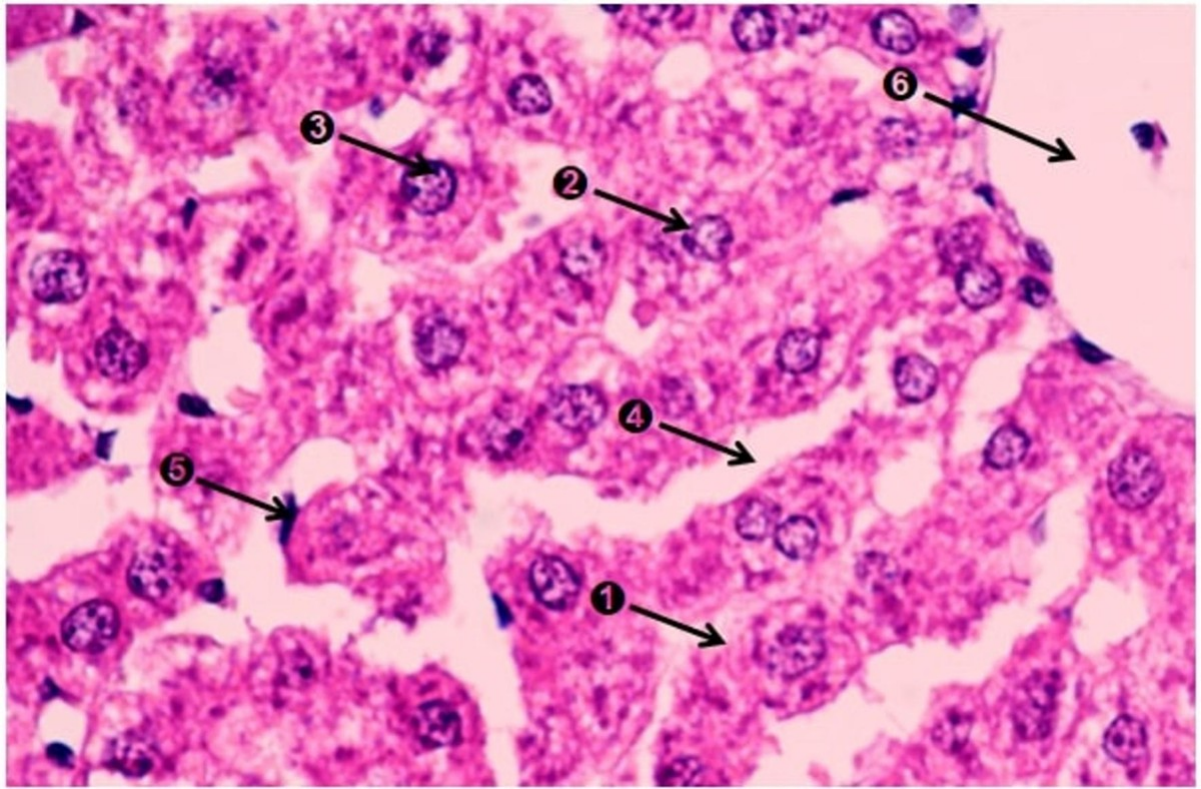
Light micrograph of rat’s liver (X400, H and E stain). 1, Hepatocytes; 2, Nuclei; 3, Nucleoli; 4, Blood sinusoids; 5, Kupffer cell; 6, Central vain.

The histological examination of rats’ livers illustrated in (Fig 6) revealed normal central vein with active nuclei, hepatocytes in polyhedral shape normal intact cell membrane with prominent nucleoli and blood sinusoids showed good caliber. Both central vein and blood sinusoids were lined by flat endothelial cells and Kupffer cells (macrophages). Safe use of ingested probiotic strains was previously reported as no free oxygen species was reported to bind with high affinity to cell membranes causing alterations in cell function [55–57].

### Application in cultured milk and probiotic yoghurt

**Fig 7:**
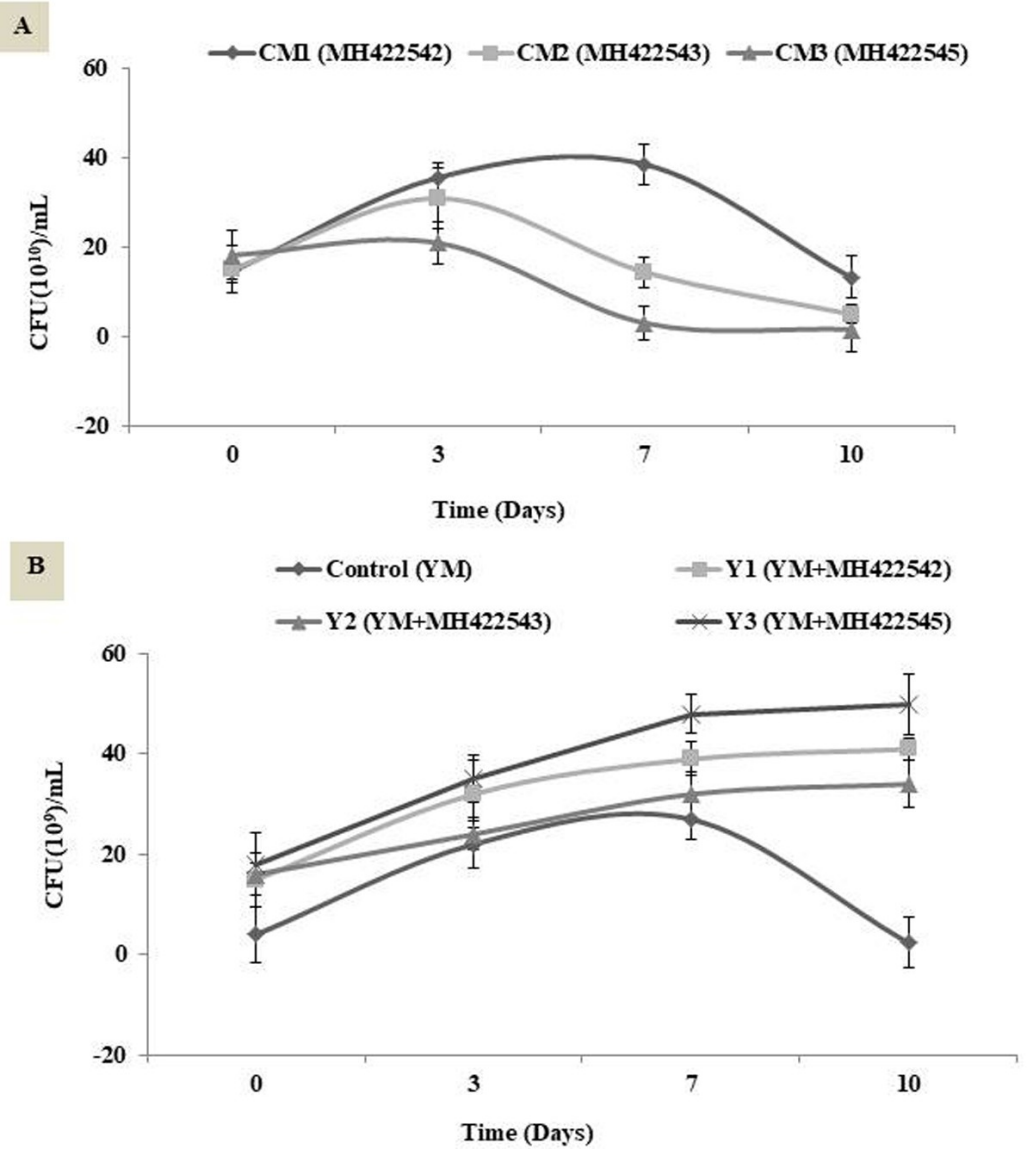
Viability of probiotic strains in cultured milk (CM) beverages and probiotic yoghurt (Y) products during storage. A, Viability of probiotic strains in cultured milk (CM) beverages; B, Viability of probiotic strains in probiotic yoghurt (Y) products. YM, Yo mix 95. MH422542, *Streptococcus thermophilus*; MH422543, *Enterococcus faecium*; MH422545, *Lactococcus lactis* subsp *lactis*.

After safety validation through *in vitro* and *in vivo* tests, the strains; *St*. *thermophilus* MH422542, *E*. *faecium* MH422543 and *Lactococcus lactis* subsp *lactis* MH422545, were applied as starter or adjunct cultures into two types of functional fermented dairy products. The viability of the single added probiotic cultures in cultured milk are illustrated in Fig 7A. All cultured milk beverages ended the 10 days experimental period with high viable count exceeded 10^10^ CFU/mL as recommended for functional foods. The variations of viability of probiotic strains in mixed cultures with yoghurt starter Yo-Mix 495 in probiotic yoghurt products, when stored at 4°C were presented in Fig 7B. The daily intake of probiotic bacteria 10^7^–10^9^ CFU/g could survive the upper ingestion to exert their positive physiological functions in the human body [1], At the end of storage the viable count in control and all pro-yoghurt products achieved the recommended count (10^7^ CFU g^-1^) in order to exert its beneficial role. The least count scored in control was 9.8 log CFU g^-1^ (7x10^9^ CFU g^-1^). These data indicated that fermenting milk by combination of probiotic and common yoghurt starter culture yielded reduction in viability of mixed strains in comparing with the fermenting using single probiotic cultures. Dave and Shah, (1998) pointed to the antagonism among the probiotic bacteria used with starter cultures caused by the production of antimicrobial substances may decrease the numbers of any sensitive organisms that may be present in a product or starter culture [58].

**Fig 8:**
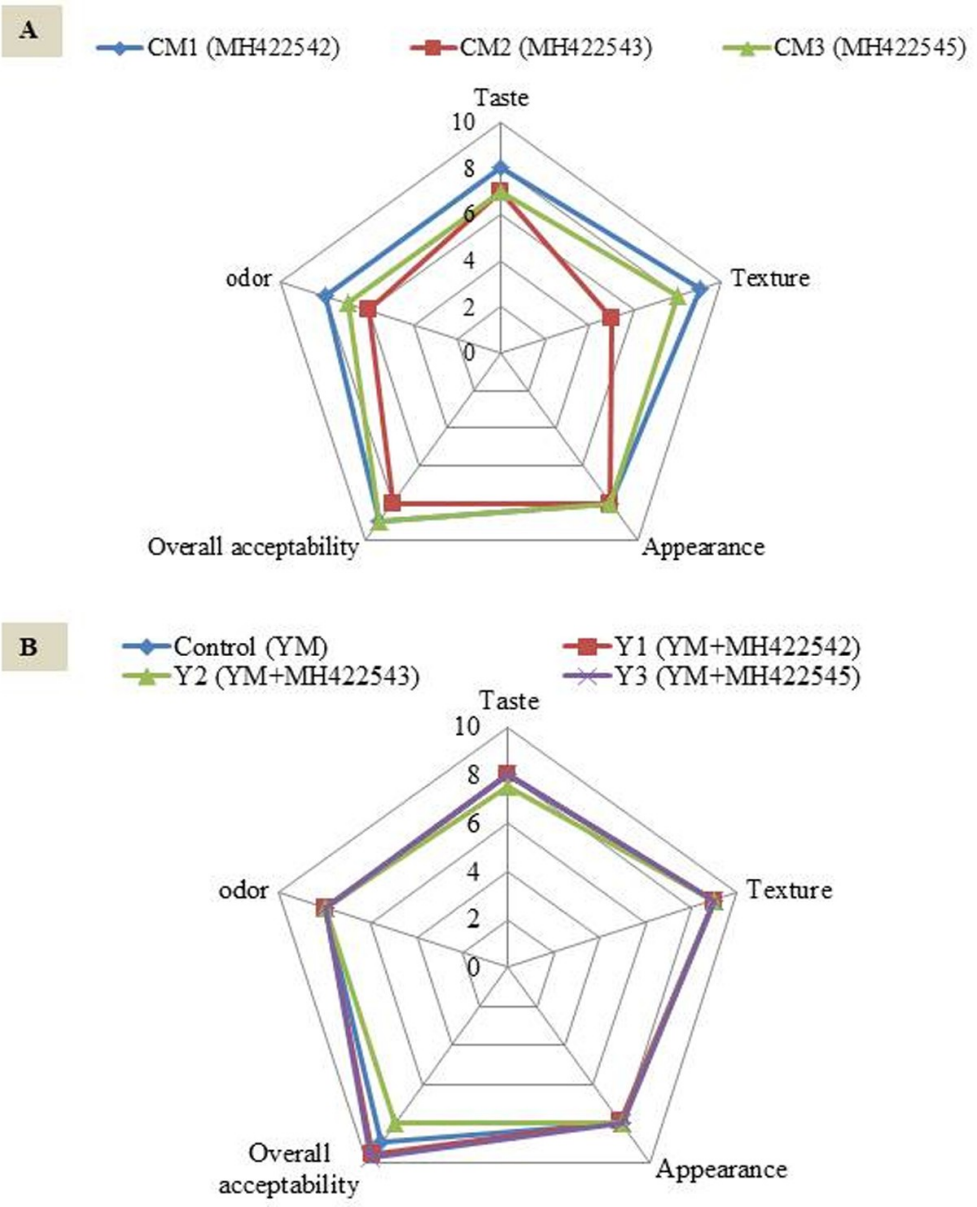
Sensory evaluation of cultured milk (CM) beverages and probiotic yoghurt (Y) products. A, Sensory evaluation of cultured milk (CM) beverages; B, Sensory evaluation of probiotic yoghurt (Y) products. YM, Yo mix 95. MH422542, *Streptococcus thermophilus*; MH422543, *Enterococcus faecium*; MH422545, *Lactococcus lactis* subsp *lactis*.

Chemical characteristics represented in pH, acidity% and synthesized acetaldehyde via metabolic activity of tested probiotic cultures during cold storage period at 4°C for 10 days of cultured milk beverages and probiotic yoghurt products are shown in Table 2. Decreased pH values was connected to the changes in viability of strains and their metabolic activity; where the increase in viable counts in day 3 and 7 accompanied with decrease in pH and vice versa in day 10, that agreed with [1]. The evaluation of aroma formation is generally based on the production of acetaldehyde, the most prominent compound in yoghurt [59, 60]. As presented in Table 2, the maximum initiating value of acetaldehyde in cultured milk products was (9.6 mg 100 mL^-1^) belonged to *St*. *therophilus* that showed significant increase on 3^rd^ day of cold storage. This could explain the yoghurt like typical flavor that described this product in sensory evaluation enhancing the taste (Fig 8). On the other hand, a significant increase in synthesizing of acetaldehyde was observed in combined probiotic cultures in probiotic yoghurt products (Table 2). These results are in consistence with Soni et al., (2020) who reported that the combination of multi-strain probiotic decreased pH, improved flavor and texture. The highest levels of acetaldehyde were achieved on culturing milk by *St*. *thermophilus* MH422542. *St*. *thermophilus* was reported to produce lactic acid, acetaldehyde and acetate as additional end-products [5].

**Table 2:** Chemical properties of cultured milk (CM) beverages and probiotic yoghurt (Y) products.

Products	Storage period (days)	pH	%Acidity	Acetaldehyde
**Cultured milk products**				
CM1 (MH422542)	0	4.41±0.02^c^	0.71±0.07^c^	9.662±1.16^f^^g^
	3	4.29±0.04^a^^b^	0.80±0.12^c^^d^	12.402±1.35^i^
	7	4.22±0.04^a^	0.85±0.09^d^	10.237±1.04^h^
	10	4.28±0.02^a^	0.84±0.04^d^	7.582±0.73^e^
CM2 (MH422543)	0	4.81±0.10^f^^g^	0.62±0.10^a^^b^	7.792±0.46^e^
	3	4.63±0.07^e^	0.65±0.06^a^^b^	8.765±1.15^e^^f^
	7	4.85±0.05^g^	0.61±0.04^a^^b^	6.459±0.74^d^
	10	4.89±0.03^g^^h^	0.59±0.06^a^	5.306±0.15^c^
CM3 (MH422545)	0	5.15±0.02^a^	0.55±0.08^a^	4.670±0.21^b^
	3	5.05±0.03^a^	0.57±0.11^a^	6.759±0.14^c^
	7	4.97±0.06^h^	0.58±0.07^c^	6.261±0.23^c^
	10	4.78±0.07^f^	0.60±0.05^c^	3.485±0.11^a^
**Probiotic yoghurt products**				
Control (YM)	0	4.62±0.05^e^	0.67±0.13^b^	28.070±2.33^j^^k^
	3	4.47±0.04^c^	0.75±0.09^c^	33.144±1.94^k^
	7	4.47±0.02^c^	0.74±0.10^c^	50.007±12.23^l^^m^
	10	4.56±0.05^d^	0.68±0.05^b^	36.684±15.30^k^
Y1 (YM+MH422542)	0	4.51±0.03^d^	0.71±0.12^c^	34.545±4.25^k^
	3	4.48±0.06^c^	0.73±0.03^c^	48.414±9.13^l^
	7	4.38±0.04^b^^c^	0.83±0.09^d^	60.784±16.04^m^^n^
	10	4.50±0.07^d^	0.71±0.05^c^	56.346±12.07^m^
Y2 (YM+MH422543)	0	4.57±0.10^d^	0.69±0.07^c^	25.484±3.10^j^
	3	4.51±0.04^d^	0.70±0.04^c^	43.169±7.24^l^
	7	4.44±0.06^c^	0.81±0.12^d^	48.216±5.08^l^
	10	4.52±0.02^d^	0.74±0.10^c^	45.088±12.11^l^
Y3 (YM+MH422545)	0	4.66±0.05^e^	0.68±0.14^b^	25.962±7.10^j^
	3	4.65±0.07^e^	0.68±0.06^b^	32.599±10.02^k^
	7	4.51±0.04^d^	0.70±0.04^b^^c^	25.863±8.25^j^
	10	4.59±0.02^d^^e^	0.68±0.13^b^	22.599±6.14^j^

Data represented are means ±SD.

Acetaldehyde content was represented in (mg/100mL).

YM, Yo mix 95.

^a,b,c,…^Means values in the same column marked with unlike letters are significantly different (*p*<0.05).

MH422542, *Streptococcus thermophilus*; MH422543, *Enterococcus faecium*; MH422545, *Lactococcus lactis* subsp *lactis*.

Fig 8A and 8B illustrated the organoleptic properties of cultured milk beverages and probiotic yoghurt products. According to overall grade scores, the product of probiotic strain; *St*. *thermophilus* (Fig 8A) gained the highest overall scores compared with other culture milk products. Flavor of yoghurt is supported by various compounds, in which lactic acid represents as the major contributor among other aroma compounds [61]. Fig 8B showed the organoleptic properties of probiotic yoghurt products; the acidic flavor dominated other flavors. On the other hand, the EPS produced by *St*. *thermophilus* strain, supported the taste and texture as products were described as sweet and creamy.

All products had acceptable appearance with typical porcelain surface. These results supports that the wild LAB isolated strains when correctly employed in dairy processing as starter or adjunct cultures, would lead to developed innovative dairy products [62].

## Conclusion

After isolation, identification and *in vitro* evaluation, three probiotic strains were selected for *in vivo* assessment in rats *St*. *thermophilus* MH422542 isolated from Laben Rayeb, Zabady; *E*. *faecium* MH422543 from Zabady and *L*. *lactis* subsp *lactis* MH422545 from mothers’ breast milk. The *In vivo* examination confirmed safety of the isolated strains in addition to functional properties such as high performance in GIT. These strains showed good properties when applied in cultured milk and functional probiotic yoghurt products. Considering the important status as GRAS microorganisms and that LAB can be used widely in the developing of new fermented milk product, in addition to functional aspects showed by the newly isolated wild LAB strains in the raised results; their using as starter/ adjunct cultures in dairy fermentations industry can be recommended. The present study can be helpful to dairy industry in developing new probiotic products and may provide a rational for selecting a combination of probiotic strains.
